# Alcohol Consumption as a Moderator of Anxiety and Sleep Quality

**DOI:** 10.1097/jnr.0000000000000300

**Published:** 2019-05-20

**Authors:** Ke-Hsin CHUEH, Christian GUILLEMINAULT, Chia-Mo LIN

**Affiliations:** 1 RN, PhD, Associate Professor, Department of Nursing, College of Medicine, Fu Jen Catholic University; and Deputy Director of Department of Nursing, Fu Jen Catholic University Hospital, New Taipei City, Taiwan, ROC; 2 MD, DBiol, Professor, Stanford University Sleep Medicine Division, Stanford, CA, USA; 3 MD, Attending Physician, Department of Sleep Center and Chest, Shin Kong Wu Ho-Su Memorial Hospital; and Adjunct Instructor, College of Medicine, Fu Jen Catholic University, New Taipei City, Taiwan, ROC.

**Keywords:** anxiety, daily alcohol consumption, poor sleep, sleep quality

## Abstract

**Background:**

Although people who sleep poorly may attempt to relieve anxiety for better sleep quality, whether daily alcohol consumption is a factor that moderates anxiety and sleep disturbance is not known.

**Purpose:**

The aim of the study was to explore (a) the association between anxiety and sleep quality and (b) whether daily alcohol consumption acted as a moderator between anxiety and sleep quality in those who reported sleeping poorly.

**Methods:**

Eighty-four participants aged 20–80 years who reported poor sleep (Pittsburgh Sleep Quality Index > 5) in northern Taiwan were enrolled in this cross-sectional study. A structured questionnaire covering demographics (including daily alcohol consumption), level of anxiety, level of depression, and perceived sleep quality was used to collect data.

**Results:**

The participants were mostly women (72.6%). The mean age was 41.81 (*SD* = 12.62) years; 51.2%, 19.0%, 13.1%, and 14.3%, respectively, had minimal, mild, moderate, and severe anxiety. After adjusting for factors related to sleep quality using multiple regression analysis, receiving sleep therapy, consuming alcohol on a daily basis, and having anxiety were found to be predictors of poor sleep quality. Moreover, daily alcohol consumption was found to moderate the relationship between anxiety and sleep quality.

**Conclusions/Implications for Practice:**

People who sleep poorly should avoid misusing alcohol to self-treat poor sleep quality or anxiety and should instead utilize sleep hygiene education and mental healthcare. Daily alcohol consumption may be a moderator between anxiety status and sleep quality.

## Introduction

Anxiety, poor sleep quality, and alcohol consumption are issues of concern worldwide (Kenney, Lac, LaBrie, Hummer, & Pham, 2013; Mann et al., 2012). Approximately 31.3% of adults have experienced poor sleep quality (Kidwai & Ahmed, 2013). In addition, 60%–70% of patients with anxiety complain of “trouble sleeping” in terms of reporting difficulties in initiating and maintaining sleep (Papadimitriou & Linkowski, 2005; Sutton, 2014). Moreover, 47%–60% of people who consume alcohol report poor sleep quality (Brower, Hoffmann, Conroy, Arnedt, & Armitage, 2011; Zhabenko, Wojnar, & Brower, 2012). Most patients who sleep poorly and have anxiety disorders complain of difficulties initiating and maintaining sleep, waking up in the middle of the night, and unsatisfactory sleep after waking (Papadimitriou & Linkowski, 2005). As a result, 30.2% of poor sleepers in one study reported using sleep medications (Kidwai & Ahmed, 2013). Alcohol is another common method of self-treating poor sleep quality (Kenney et al., 2013; Vitiello, 1997).

Anxiety, poor sleep quality, and alcohol consumption are complex and interactive phenomena with numerous correlates, including the physical and psychosocial health of the individual, factors in the social environment, and lifestyle factors (Brower, Krentzman, & Robinson, 2011; Kenney et al., 2013; Saatcioglu, Yapici, & Cakmak, 2008). People with anxiety typically complain of difficulties in initiating and maintaining sleep (Papadimitriou & Linkowski, 2005). The positive association between anxiety and poor sleep quality is clear (Adams & Kisler, 2013; Dean et al., 2010; Hsieh et al., 2011). However, the relationship between alcohol consumption, and anxiety and sleep quality is unconfirmed. Therefore, this study was designed to investigate the relationship between anxiety and sleep quality in participants who reported sleeping poorly and to assess whether alcohol consumption moderates this relationship.

### Background

Two fifths (20.8%) of 4,065 subjects older than 15 years (95% CI [19.6, 22.1]) in one study were reported to experience insomnia symptoms at least three nights per week (Ohayon & Sagales, 2010). Sleep quality worsens with age and in women and has been associated with level of education, intimate partner status, number of children living at home, substance use status, general health status, and mental health status (Kidwai & Ahmed, 2013). People with long-term poor sleep quality have increased rates of both morbidity and mortality (Hall et al., 2015; Léger & Bayon, 2010; Rod et al., 2014). Poor sleep not only decreases quality of life but also reduces cognitive function and increases the risk of being involved in an accident (Avidan et al., 2005; Karimi et al., 2013; Yaffe, Falvey, & Hoang, 2014).

Although alcohol is a sedative and may induce the rapid onset of sleep, the resultant disturbance of nighttime sleep quality and of respiration may result in postconsumptive daytime impairment (Vitiello, 1997). In one study, 75%, 69%, and 52% of alcohol drinkers, respectively, complained of waking early, experiencing difficulties in maintaining night sleep, and experiencing difficulties initiating and maintaining sleep (Foster, Peters, & Kind, 2002). Moreover, people with high levels of alcohol consumption revealed decreased slow-wave activity during non-REM sleep (Brower, Hoffmann, et al., 2011). Thus, 30% of alcohol drinkers were found to rely on taking hypnotic tablets to induce sleep (Foster et al., 2002). In addition to sleep health consequences, the comorbidity of anxiety and alcohol use disorders has long been recognized (Mann et al., 2012; Saatcioglu et al., 2008).

Anxiety is one of the most common psychiatric disorders, creating an enormous burden both on the afflicted and on the society (Mann et al., 2012). On the basis of a meta-analysis, 177 studies with data from 7,151 psychiatric patients, including those experiencing anxiety disorders, showed significantly reduced total sleep time and sleep efficiency (SE) and prolonged sleep latency (Kupfer & Reynolds, 1992; Kyle et al., 2014). One study reported that alcohol consumption decreased the risk of anxiety (Mann et al., 2012). However, 40% and 33% of people with alcohol consumption were found to have “some” or “extreme” anxiety problems, respectively (Boschloo et al., 2013; Foster et al., 2002).

The effects of alcohol consumption on sleep quality are complex and interactive (Marmorstein, 2017; Miller et al., 2017). This study sought to investigate the relationships among anxiety, poor sleep quality, and alcohol.

### Purpose

This study used a cross-sectional survey to (a) investigate the association between anxiety and sleep quality in participants who reported poor sleep and (b) evaluate the moderating effect of daily alcohol consumption on this association.

## Methods

### Design and Ethical Issues

The institutional review board of the target hospital and university (no. 20111101R) approved this study in Taiwan for 2012–2013. Informed consent was obtained from each participant. Each participant took approximately 10–15 minutes to complete the questionnaire.

### Participants

Individuals 20 years and older who complained of poor sleep but had no cognitive limitations, inability to communicate, or unstable vital signs were recruited from a medical center sleep clinic and a university. On the basis of the strategy used in previous studies (Y. H. Lin, Chueh, & Lin, 2015) to estimate sample size (Rosner, 2015), the estimated minimum sample size in this study was set at 76 to avoid statistical problems in regression analysis under an effect size of 0.19, α of .05, and β of .2. With a 10% attrition rate taken into consideration, 83 participants were required.

### Instruments

A structured questionnaire including four parts was used to conduct face-to-face interviews with the participants. This questionnaire consisted of four parts, which are discussed below.

The first section gathered data on respondent age (years), gender (male or female), sleep therapy (visiting the sleep disorder clinic), college education, occupation, intimate partner status, having at least one child, having good general health, daily consumption of alcohol or caffeine, and daily use of tobacco or sedative medicines.

The second part gathered level-of-anxiety information using a revised version of the Chinese version of the Beck Anxiety Inventory (BAI), a 21-item, self-report inventory for measuring severity of anxiety in psychiatric populations. The responses are rated on a scale ranging from 0 = *not at all* to 3 = *severely/unbearably* for each symptom of anxiety over the past week. Total scores ranged from 0 to 63, with a higher score representing more severe anxiety. Total BAI scores of 0–7, 8–15, 16–25, and 26–63 are respectively interpreted as “minimal,” “mild,” “moderate,” and “severe” anxiety. The original BAI showed high internal consistency (α = .92) and test–retest reliability (*r* = .75; Beck, Epstein, Brown, & Steer, 1988; Y. J. Lin, 2000). The Cronbach's α for this study was .95, and the content validity index (CVI) was 1.00.

In addition, the Chinese version of the Beck Depression Inventory-II (BDI-II) was used to assess level of depression. The BDI-II has 21 items, each of which consists of four self-evaluative statements that are scored from 0 to 3, with higher scores indicating greater depression severity. Responses are summed to yield a total score ranging from 0 to 63. The original BDI showed high internal consistency (α = .91) and test–retest reliability (*r* = .93; Beck, Steer, Ball, & Ranieri, 1996; Chen, 2000). The Cronbach's α coefficient for the Chinese-version BDI-II in this study was .97, and the CVI was 1.00.

Finally, the Chinese version of the Pittsburgh Sleep Quality Index (PSQI) was used to measure sleep quality during the past month using data on sleep latency (minutes), total sleep time (hours), time in bed (hours), and SE (%). The PSQI includes the seven components of subjective sleep quality, sleep latency, sleep duration, SE, sleep disturbance, use of sleep medication, and daytime dysfunction, with each component calculated separately. Item scores range from 0 to 3 points, and the total possible score ranges from 0 to 21 points, with higher scores associated with poorer quality of sleep. The 5-point cutoff has a sensitivity of 89.6%, a specificity of 86.5%, and a correct sleep disturbance diagnosis rate of 88.5%. The internal reliability consistency and test–retest reliability of the original PSQI were .83 and .85, respectively (*p <* .001; Buysse, Reynolds, Monk, Berman, & Kupfer, 1989). In this study, participants who earned a PSQI score above 5 were identified as sleeping poorly. The Cronbach's α for this study was .64, and the CVI was 1.00.

### Statistical Method

Data analysis was performed using IBM SPSS Statistics for Windows, Version 20.0 (IBM, Armonk, NY, USA). Descriptive statistics were used to describe the demographic characteristics of participants. A Student's *t* test or Pearson correlation was used to analyze the association between participants' sleep quality and demographic characteristics.

In this study, “participants with daily alcohol consumption” were coded as 1 and “participants without daily alcohol consumption” were coded as 0. These two values were then used to deal with the terms of interaction for daily alcohol consumption levels and anxiety scores. If the hypothesized moderator (daily alcohol consumption) was significantly associated with the level of sleep quality in the multiple regression analysis, the interaction (Daily Alcohol Consumption × Score of Anxiety) was added to the multiple regression model to examine the moderating effect.

Finally, if the interaction was found to be significantly associated with sleep quality in the multiple regression analysis, the interaction between anxiety and sleep quality for those participants with and without daily alcohol consumption was calculated. A two-tailed *p* value of less than .05 was considered statistically significant.

## Results

### Participant Characteristics

The mean for age for the 84 participants in this study was 41.81 years. Slightly more than half (*n* = 47, 56%) were recruited from a hospital sleep disorder clinic, and the remaining 37 (44%) were recruited from a university. The mean scores for anxiety, depression, and sleep quality were 12.24, 14.67, and 12.13, respectively, and 51.2%, 19.0%, 13.1%, and 14.3% had minimal, mild, moderate, and severe anxiety, respectively. Furthermore, 7.1% consumed alcohol on a daily basis. Associations between sleep quality and demographic characteristics included age (*p* = .004), gender (*p* = .002), sleep therapy (*p* < .001), college education (*p* = .002), occupation (*p* = .003), good general health (*p* = .010), daily caffeine consumption (*p* = .037), daily sedative medicine use (*p* = .001), anxiety (*p* < .001), and depression (*p* = .001; see Table 1).

**TABLE 1.**
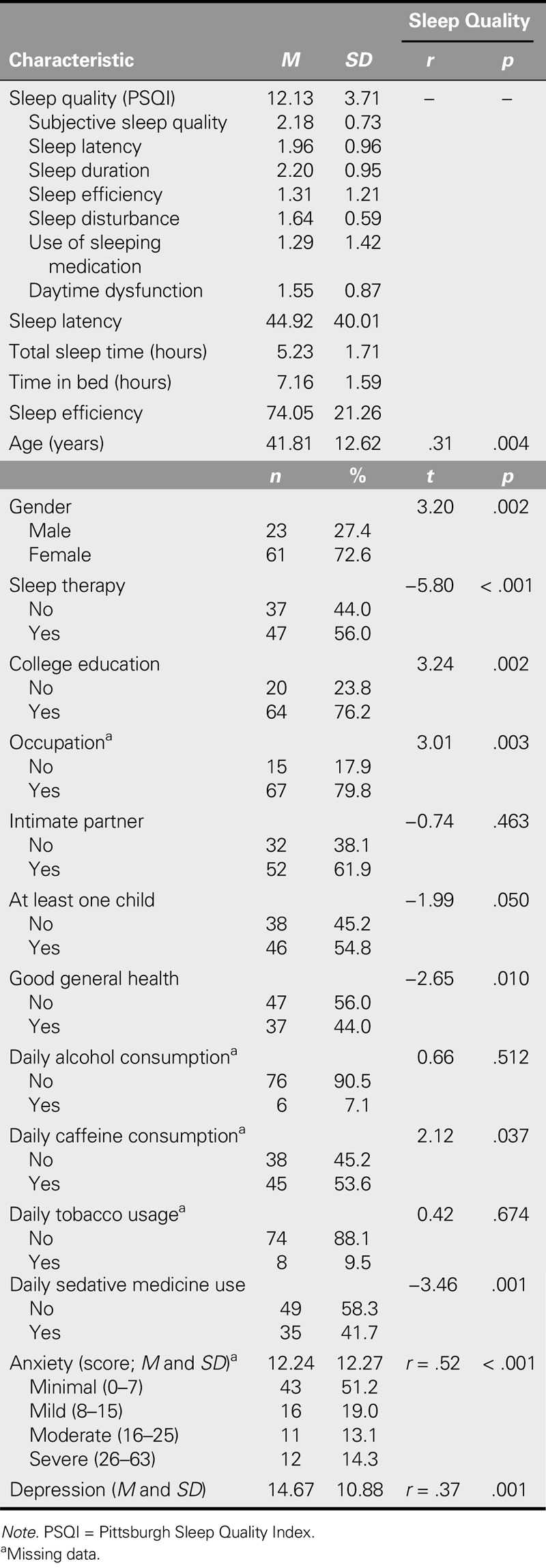
Distributions and Correlates of Level of Sleep Quality: Unit Characteristics (*N* = 84)

After adjusting for the effects of demographic characteristics, participants who reported sleeping poorly, were currently participating in sleep therapy, consumed alcohol daily, or perceived a higher level of anxiety reported poorer sleep quality than those who did not, *F*(14, 61) = 6.48, *p* < .001 (Model 1 in Table 2). Model 1 variables had a significant association of 51% with poor sleep quality. The interaction between daily alcohol consumption and anxiety was selected for regression analysis, *F*(15, 60) = 6.76, *p* < .001 (Model 2 in Table 2). Model 2 variables had a significant association of 54% with poor sleep quality. The results indicate that the interaction between daily alcohol consumption and anxiety scores (*t* = 2.21, *p* = .031) was significantly associated with sleep quality.

**TABLE 2.**
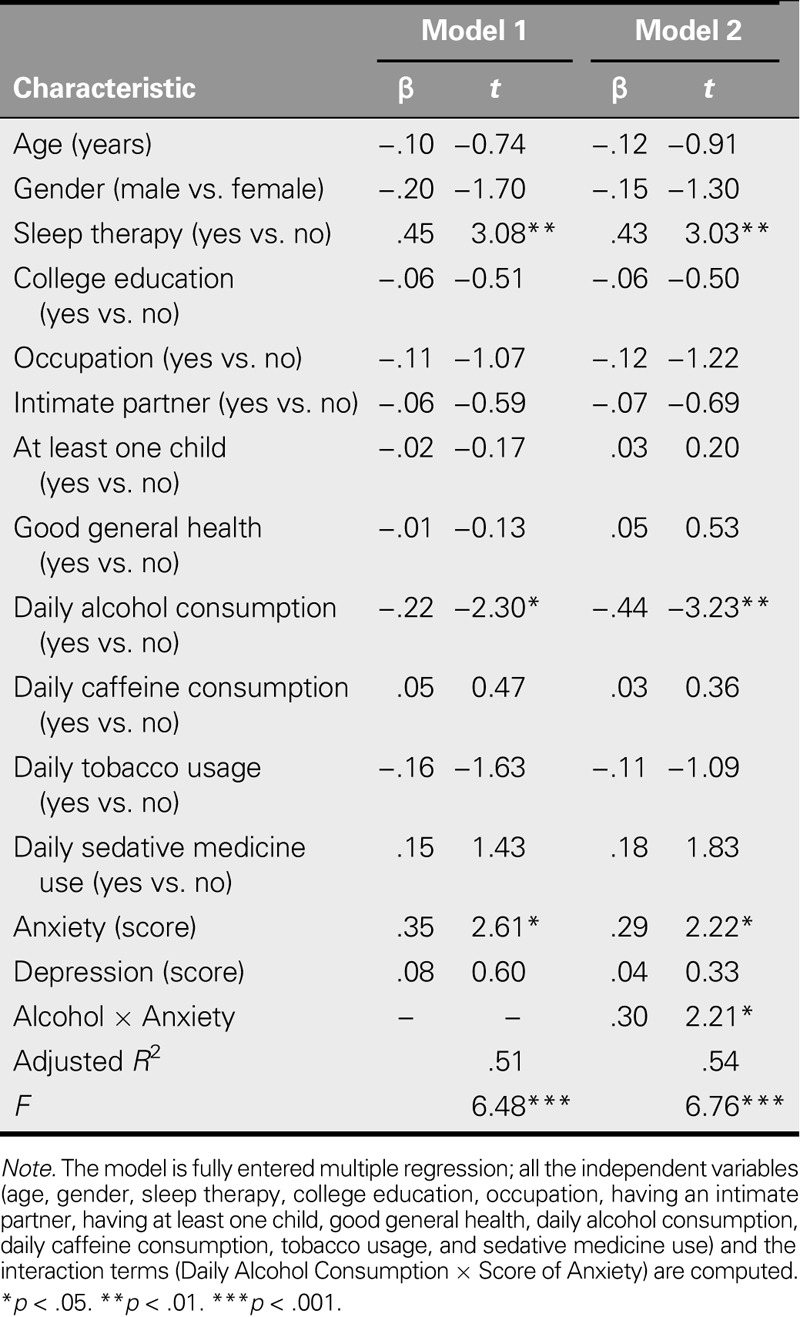
Correlates of Sleep Quality: Multiple Regression Analyses (*N* = 84)

**Figure 1.**
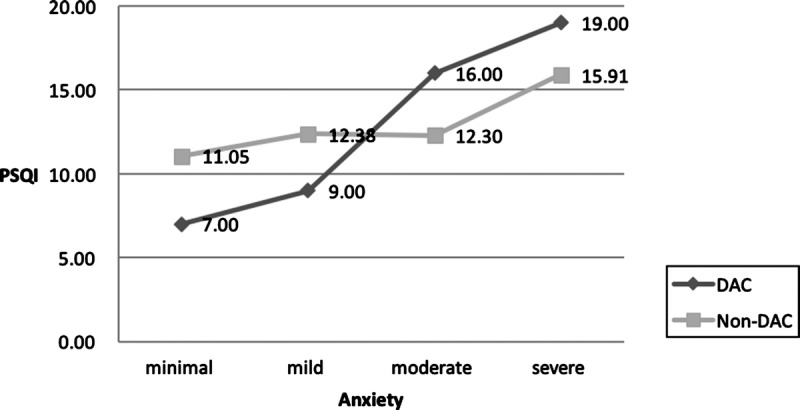
Association between sleep quality (Pittsburgh Sleep Quality Index [PSQI] score) and daily alcohol consumption (DAC) in poor sleepers with minimal, mild, moderate, and severe anxiety (*N* = 84).

Furthermore, the results highlighted a significant difference in quality of sleep between participants with minimal, mild, moderate, and severe anxiety who did consume alcohol on a daily basis and their counterparts who did not (Figure 1). Both lines intersect at a point between mild and moderate PSQI-determined levels of anxiety.

## Discussion

The participants in this study were mostly women (72.6%). The mean age of participants was 41.81 ± 12.62 years. The average score for sleep quality was 12.13 ± 3.71, which is similar to another study in Taiwan of a population of women who reported sleep disturbances (Chueh & Chang, 2014). In this study, 48.8% of the participants experienced at least a mild level of anxiety; although this prevalence is similar, the rate of severe anxiety found in this study is higher than that of a study by Y. H. Lin et al. (2015). The negative association between anxiety and sleep quality found in this study is similar to results from a study by Benca, Obermeyer, Thisted, and Gillin (1992).

This study distinguished between participants who consumed alcohol daily and those who did not and then investigated whether daily alcohol consumption had a moderating effect on the association between anxiety and sleep quality. According to the selected criteria, moderation occurs when the interaction between the factor (score of anxiety) and the hypothesized moderator (daily alcohol consumption) is significantly associated with the dependent variable (score of sleep quality) after controlling for the buffering effects of other factors. Although many studies have shown daily alcohol consumption and anxiety to be the main predictors of poor sleep quality (Brower, Hoffmann, et al., 2011, Brower, Krentzman, et al., 2011; Zhabenko et al., 2012), this study further highlights that consuming alcohol on a daily basis may have a moderating effect on the relationship between anxiety and sleep quality. In this study, participants who reported minimal or mild anxiety and who consumed alcohol on a daily basis tended to have better sleep quality. However, participants with moderate or severe anxiety who consumed alcohol on a daily basis tended to have worse sleep quality (Figure 1). Consequently, although alcohol may improve sleep quality among those with minimal and mild levels of anxiety by decreasing sleep latency, it may also disturb nighttime sleep quality and respiration, resulting in mostly Stage 1–2 (non-REM) sleep patterns (Vitiello, 1997). Therefore, to prevent the misuse of alcohol to self-treat poor sleep quality or anxiety, clinical professionals should pay attention to potential interactions between daily alcohol consumption and anxiety in patients who report poor sleep.

### Conclusions

Daily alcohol consumption was shown to moderate the relationship between anxiety status and sleep quality among participants who slept poorly. Although daily alcohol consumption may improve sleep latency in those with minimal to mild levels of anxiety, it may decrease sleep quality in those with severe anxiety. Therefore, alcohol should not be used as a remedy for poor sleep quality or anxiety; instead, patients should seek cognitive behavior therapy and mental healthcare for greater therapeutic efficacy.

### Limitations

There are several limitations to this study. First, only 7.1% of the participants reported both poor sleep and daily consumption of alcohol. This may limit the generalizability of the findings. Second, this study asked only whether participants consumed alcohol on a daily basis. To better elucidate the effect of alcohol consumption on sleep, additional information on drinking should be gathered, including pattern and time of day information. Third, because both sleep quality and anxiety are subjective experiences, these factors likely interact with each other easily. We suggest further research using objective sleep and anxiety data such as heart rate variability to obtain a more comprehensive picture of the sleep quality and anxiety status of participants.
